# Implementation fidelity of provider-initiated HIV testing and counseling of tuberculosis patients under the National Tuberculosis Control Program in Kathmandu District of Nepal: an implementation research

**DOI:** 10.1186/s12913-019-4343-3

**Published:** 2019-08-02

**Authors:** Randeep Kumar, Ari Probandari, Biwesh Ojha, Ashmin Hari Bhattarai, Yanri Wijayanti Subronto

**Affiliations:** 1 Department of Public Health and Nursing, Universitas Gadjah Mada, Jl. Farmako Sekip Utara, Yogyakarta, 55281 Indonesia; 2 Department of Public Health, Universitas Sebelas Maret, Surakarta, Indonesia; 3 Hope International College, Affiliated to Purbanchal University, Kathmandu, Nepal; 4 Dept of Internal Medicine/Center for Tropical Medicine, Faculty of Medicine, Publih Health, and Nursing, Universitas Gadjah Mada, Jl. Farmako Sekip Utara, Yogyakarta, Indonesia

**Keywords:** Implementation Fidelity, TB, HIV, PITC, DOTS, Nepal

## Abstract

**Background:**

There exists low uptake of Human Immunodeficiency Virus (HIV) testing among Tuberculosis (TB) patients through Provider-Initiated HIV Testing and Counseling (PITC) under the national TB control program in Nepal. The degree and quality of program delivery were explored through determining whether the PITC program is currently implemented as intended. This study aimed to assess three major components of the program’s implementation fidelity: adherence to PITC service, exposure, and quality of program delivery in order to optimize and standardize PITC implementation by exploring its barriers and enablers.

**Methods:**

This research used a sequential explanatory mixed method design. Retrospective cross-sectional study of TB patients enrolled in five TB treatment centers of the Kathmandu district from July 1, 2016, to June 30, 2017 was done to assess PITC adherence to Direct Observed Treatment-Short Course (DOTS) protocols. The centers’ TB-DOTS readiness was assessed using the WHO Service Availability and Readiness Assessment checklist. A qualitative study was conducted to explore the barriers and enablers of PITC service implementation.

**Results:**

From a total of 643 TB patients registered, 591 (92.1%) patients were offered HIV test counseling. Amongst those, 571 (96.6%) accepted and 523 (91.5%) were tested. Service providers’ HIV knowledge was found to be good although only 2/5 (40%) had participated in PITC training. The key barriers experienced by service providers were: patients feeling offended, stigmatization and lack of human resources in DOTS centers. The main enablers for PITC were national TB program commitment, health workers’ motivation, collaboration between stakeholders and external development partners’ promotion of program implementation.

**Conclusion:**

In the selected study sites, PITC services are well integrated into the routine TB control program with a high uptake of HIV testing among registered TB patients. This achievement should be sustained by addressing the identified barriers mainly in the quality of the PITC program delivery.

**Electronic supplementary material:**

The online version of this article (10.1186/s12913-019-4343-3) contains supplementary material, which is available to authorized users.

## Background

Tuberculosis (TB) is the 9th leading cause of mortality and one of the primary causes of death for patients with Human Immunodeficiency Virus (HIV) worldwide [1]. In 2017, there were estimated 1.3 million TB-related deaths among HIV-negative people and an additional 300,000 deaths due to TB among HIV-positive people, according to the World Health Organization (WHO) Global TB Report 2018. Globally, people living with HIV (PLHIV) are on average 21 times (16–27) more likely to develop active TB [2]. TB/HIV co-infection is increasing in Southeast Asia. Four (Thailand, Myanmar, India, Indonesia) out of eleven countries have high TB/HIV burden in this region ranging from 2 to 16% [3].

A TB/HIV situation analysis in Nepal was initiated in 2006 to facilitate the development of common policies and strategies for the effective implementation of TB/HIV collaborative activities. To take advantage of this situation analysis on the strengths and to address the weaknesses of both programs, the strategic policy guidelines on collaborative TB/HIV activities in Nepal was developed in 2008. To convert policy into action, the Implementation Guidelines on TB and HIV/AIDS collaboration in Nepal was introduced in the year 2009. The National Tuberculosis Center (NTC) and National Center for AIDS and STI Control (NCASC) agreed to work in collaboration to strengthen the TB and HIV collaborative activities at all levels. As per the national PITC guidelines, HIV tests are offered routinely to all TB patients registered in the Directly Observed Treatment, Short-course (DOTS) and those who are found seropositive are referred to the Antiretroviral Therapy (ART) center for assessment and initiation of ART course [4].

The WHO interim policy on TB/HIV co-infection recommends Provider-Initiated HIV Testing and Counselling (PITC) services for TB patients as an entry point to integrated health care services with the aim to decrease the incidence and treat both TB and HIV infections [5]. Evidence shows that uptake of HIV tests is higher in many countries through PITC programs among TB patients [6–8], however, in Nepal uptake of HIV tests among TB patients is only 17% and reached around 20% in the Kathmandu district in the fiscal year 2015–16 [9, 10]. The implementation fidelity of PITC program has never been assessed in Nepal.

### Implementation fidelity framework

Fidelity is well-defined as the degree to which an intervention was implemented as recommended in the approved protocol or as it was proposed by the program designers [11]. There are five major fidelity dimensions tested when considering program fidelity: i.e. adherence, exposure, facilitation strategies, quality of program delivery and participant responsiveness or involvement among them. We adopted three common dimensions: adherence to PITC service, exposure and quality of program delivery in order to optimize and standardize PITC implementation and further explore its barriers and enablers [11–13]. Adherence refers to “all principal components being delivered to the correct population; staff trained properly; using the specific protocols, procedures, and materials; and in the contexts approved” [11, 12]. Exposure refers to “the frequency through which program packages’ techniques were applied” [11, 12]. Quality of program delivery refers to “assessment of the manner of health workers deliver program interventions (intervention techniques skills or approaches prescribed by the guideline, interest, readiness, and attitude)” [11, 12]. Moreover, rather than seeing each of these dimensions as an alternative measure, Carroll et al. conceptualized adherence to include content, frequency, duration and coverage as the main measurement of implementation fidelity and the other dimensions as moderators [11]. This study aimed to assess the implementation fidelity along with the barriers and enablers to the PITC among TB patients under the national TB control program in one of the highest TB and HIV prevalence districts of Nepal.

## Methods

This study followed the Standards for Reporting Implementation Studies (StaRI) guidelines for methodology and reporting the results [14].

### Study design

This study used a mixed method explanatory sequential design, which involved quantitative study followed by qualitative study, and adapted the fidelity framework of Carroll [11]. Quantitative study covered two different parts: the first was access to the TB register to review HIV testing and counseling (HTC) offered from service providers and patients’ responses towards PITC services (TB patients, Offered, Accepted, and HTC attended), and the second was assessement of service availability and readiness of DOTS centers’ PITC services through structured questionnaires (WHO, 2014b). Qualitative study included in-depth interviews and key informant interviews to identify the barriers and enablers to implementation fidelity of the PITC program.

### Study setting

This study was conducted in Kathmandu district of Nepal, which has reported high numbers of notified TB patients (3298), and has the highest case notification rate (160) in Nepal. It is also one of the 13 high burden TB districts in Nepal [10]. Thus, Kathmandu was purposefully selcted for this study.

To balance logistic feasibilty with time constrains, five DOTS centers were purposefully selected in coordination with the District Public Health Office (DPHO) team after an initial consultation meeting, taking into consideration: (i) High load of TB patients, (ii) DOTS centers’ accessibility, and (iii) Similarity in the provision of services since 3 out of 5 study sites act as referral centers in the country. The 5 selected sites included one private referral center managed under non-governmental organizations (TB-DOTS center A), two TB treatment outlets at community hospitals (TB-DOTS centers B and C), and two public referral centers (TB-DOTS centers D and E). The selected hospitals are tertiary care centers with provision of specialized services being delivered to patients.

### Data collection and analysis

The quantitative data were collected from the Health Management Information system (HMIS) TB treatment register of the national TB control program. The major function of HMIS in Nepal is to collect and manage the health service delivery information for all levels of health service delivery outlets. Data of all registered TB patients under the National TB control program from July 01, 2016 to June 30, 2017, in the five selected TB treatment outlets (TB-DOTS Centers A-E) were collected from the HMIS TB register. The period from July 01, 2016 to June 30, 2017, includes one complete fiscal year 2074/2075 Bikram Sambat (BS) in Nepal. A secondary data extraction tool (Additional file 1) was developed to retrieve data on health workers’ adherence and TB patients’ responsiveness towards PITC services from selected facilities. Data of TB control program routine records, referral forms of TB-DOTS and ART centers’ records were used. Data on patients sociodemographic characteristics, TB types, TB categories, TB treatment, HIV test offered, HIV test accepted, HIV test attended, HIV results, referral to ART centers and evaluation of initiation of ART services are recorded by service providers in the HMIS TB register during patients’ visits and those details were extracted from the HMIS TB register.

The WHO Service Availability and Readiness Assessment checklist was modified to collect data on delivery quality of PITC services from each DOTS center (Additional file 2) [15]. Data on DOTS related information, DOTS human resources status, type of TB/HIV co-infection services provided, DOTS patient load, reporting and supervision information, services provided along with equipment and supplies availability were collected. This checklist was administered to service providers with additional observations to verify information as needed. The service availability and readiness component scores depend upon the availability of the respective component domains. The mean availability of domain items is calculated as a total number of available domain items for each component and divided by the total of domain items, multiplied by 100 for a percentage score. The achieved percentage was categorized into three groups:  minimum to 39 % = Poor readiness, 40 % to 79 % = Acceptable readiness and 80 % to maximum = Good readiness [15]. Data were entered on EpiData v3.1 and later exported for analysis to STATA 13 (STATA Corp., 2013). Mean and standard deviation (SD) values were computed for continuous variables and frequencies for categorical variables.

Prior appointments were made with service providers of the five selected DOTS centers for in-depth interviews (IDIs) and with program managers for key informant interviews (KIIs). Five IDIs were conducted with service providers and two KIIs with district managers of DPHO. An interview guide was developed to facilitate both IDIs and KIIs (Additional file 3). This guide was developed to determine the barriers and enablers to implementation fidelity of the PITC program and included key areas, such as Management, Workload of staff, Coordination, Ownership, HIV test, Standard Operating Procedures (SOP), Logistic Supply, and Related training.

All IDIs and KIIs were recorded and completely transcribed in Nepali and then translated into English. After transcription, two phases of data validation were performed; firstly, co-authors checked the transcriptions with recorded audio files, and this process was repeated for rechecking to ensure accuracy. Secondly, a preliminary analysis was shared with stakeholders, feedback was collected and dominant themes compared to determine the areas of agreement as well as disagreement for data triangulation.

After reviewing the transcripts, themes and corresponding codes were developed for further data organizing and analyzing. This process was done in OpenCode 4.03 software. Identification of themes and coding allowed the researchers to identify and assess the enablers and barriers to implementation fidelity of PITC through service providers’ and program managers’ perceptions and experiences.

## Results

### Patients’ characteristics

A total of 643 TB patients registered for TB treatment from July 01, 2016 to June 30, 2017, were included in this study. Nearly one-third of the respondents (30%) were from TB-DOTS center A. The mean age of patients was 33.4 and their ages ranged from 1 to 98 years. Further details of patients’ characteristics are provided in Table 1.

**Table 1:** TB patients characteristics by TB-DOTS centres (*n* = 643)

Variable		All	TB-DOTS Centre’s^a^				
		n (%)	A n (%)	B n (%)	C n (%)	D n (%)	E n (%)
Total Registered TB Cases		643 (100)	193 (30)	154 (24)	126 (20)	64 (10)	106 (16)
Sex	Male	347 (53.8)	103 (53.4)	84 (54.5)	64 (50.8)	38 (59.4)	57 (53.8)
	Female	297 (46.2)	90 (46.6)	70 (45.5)	62 (49.2)	26 (40.6)	49 (46.2)
Age Group (years)	0–14	34 (5.3)	11 (5.7)	12 (7.8)	4 (3.2)	2 (3.1)	5 (4.7)
	15–24	206 (32)	59 (30.6)	39 (25.3)	56 (44.4)	13 (20.3)	39 (36.8)
	25–49	284 (44.2)	81 (42)	74 (48)	52 (41.2)	37 (57.8)	40 (37.7)
	Above 50	119 (18.5)	42 (21.8)	29 (18.8)	14 (11.1)	12 (18.8)	22 (20.7)
Ethnic Group^b^	Dalit	15 (2.3)	5 (2.6)	2 (1.3)	1 (0.8)	5 (7.8)	2 (1.9)
	Janajati	375 (58.3)	107 (55.4)	77 (50)	102 (81)	33 (51.6)	56 (52.8)
	Madhesi	26 (4.1)	7 (3.6)	5 (3.2)	2 (1.6)	7 (10.9)	5 (4.7)
	Muslim	9 (1.4)	3 (1.6)	3 (2)	1 (0.8)	1 (1.6)	1 (0.9)
	Brahman/Chhetri	218 (33.9)	71 (36.8)	67 (43.51	20 (15.8)	18 (28.1)	42 (39.6)

^a^A = Private referral hospital, B & C = Community hospital, and D & E = Public referral hospital

^b^Ethnic group as classified by the, Health Management and Information System of Nepal

### PITC services’ adherence and exposure

**Fig. 1:**
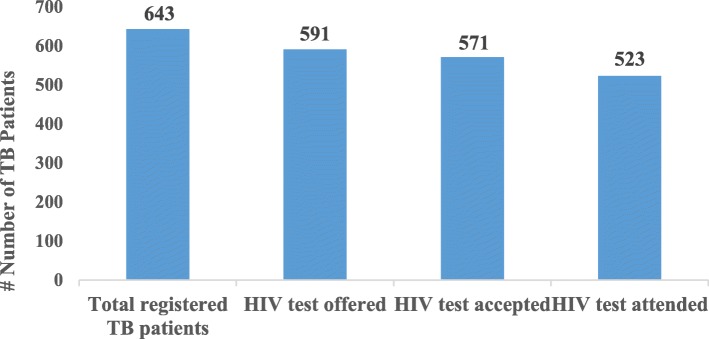
Outcome of PITC services for registered TB patients

Adherence and exposure to PITC services was determined through uptake of HIV testing and ART referral. As intended by the program, service providers initiated and offered HIV testing and counseling to registered TB patients, and most of them accepted and attended the tests. The majority of TB patients who were offered PITC services attended HIV testing services through PITC program, namely 91.5% (523/591). Among the attendees, 22 were HIV infected. All of the HIV infected TB patients were referred for ART (22, 100%) (Table 2). The PITC outcomes are presented in Fig. 1.

**Table 2:** Summary of PITC services outcome by DOTS centres

Variable		All	DOTS Centres^a^				
		n (%)	A n (%)	B n (%)	C n (%)	D n (%)	E n (%)
HIV test Offered (n=643)	Yes	591 (91.9)	191 (98.9)	152 (98.7)	82 (65.1)	63 (98.4)	103 (97.2)
	No	52 (8.1)	2 (1.1)	2 (1.3)	44 (34.9)	1 (1.6)	3 (2.8)
HIV test Accepted (n=591)	Yes	571 (96.6)	183 (95.8)	147 (96.7)	80 (97.6)	61 (96.8)	100 (97.1)
	No	20 (3.4)	8 (4.2)	5 (3.3)	2 (2.4)	2 (3.2)	3 (2.9)
HIV test Attended (n=571)	Yes	523 (91.6)	167 (91.4)	117 (79.6)	80 (100)	60 (98.4)	99 (99)
	No	48 (8.4)	16 (8.6)	30 (20.4)	0	1 (1.6)	1 (1)
HIV test Results (n=523)	Positive	22 (4.2)	2 (1.1)	3 (2.6)	1 (1.2)	15 (25)	1 (1)
	Negative	501 (95.8)	165 (98.9)	114 (97.4)	79 (98.8)	45 (75)	98 (99)

^a^ A= Private referral hospital, B & C= Community hospital, and D & E= Public referral hospital;

The main barriers perceived by service providers of DOTS centers B and C regarding PITC services were social stigmatization, highly educated and rich patients not accepting the test, rejecting the test due to fear of knowing their HIV status, difficulty to offer the test to children and elder ages by services providers, and test is voluntary so it cannot be forced onto patients. Details about the barriers are described below in the barriers section.

### Quality of program delivery in TB-DOTS centers

All five TB-DOTS centers are located in the center of the metropolitan city of Kathmandu. TB-DOTS centers A and D are specialized hospitals for TB treatment and management. These TB-DOTS centers are equipped with only one paramedic health worker who is responsible for all TB-DOTS related work (treatment and patient management). However, the TB-DOTS centers B and E also expressed their difficulties in operating day to day work with only one staff person.

*“....I am the only staff here to operate all DOTS services and its management. I am busy the whole day regulating the daily DOTS service and maintaining recording/reporting so, I am not able to bring commodities from DPHO sometimes....”* (*Health Workers, TB-DOTS centre)*

Overall, three out of five (60%) TB-DOTS centers were in the good category in terms of basic amenities. Only two TB-DOTS centers had a private consultation room for PITC services while all TB-DOTS centers have a designated toilet facility for their patients.

Good infection and prevention control measures were observed in three TB-DOTS centers. These included open space areas for waiting, open windows and ceiling fans for ventilation. The majority of TB patients came wearing a facemask in each of the centers and those patients who came without a facemask were instructed to put one on by service providers. However, service providers were only using normal facemasks during TB service delivery.

All TB-DOTS centers offer HIV testing and/or refer to the nearest HTC centres. None of the TB-DOTS centers received the national guideline of the PITC program. TB treatment outlets were not physically connected with the HIV counselling and testing clinic. Inadequate space size with poor physical conditions was observed in all TB-DOTS centers. The overall service availability and readiness status of TB-DOTS centers are presented in Table 3.

**Table 3:** The overall status of PITC service by DOTS centres

Overall Status by each Components	DOTS Centres^a^				
	A	B	C	D	D
Basic Amenities	**+++**	**+++**	**+++**	**+++**	**+++**
Basic Equipment	**+++**	**++**	**+++**	**++**	**++**
Infection and Prevention	**+++**	**++**	**++**	**++**	**++**
Information System	**+++**	**++**	**++**	**++**	**++**
PITC Service	**++**	**++**	**++**	**++**	**++**

“**+++**” = Good Readiness, “**++**” Acceptable Readiness, “**+**” Poor Readiness

^a^ A= Private referral hospital, B & C= Community hospital, and D & E= Public referral hospital;

### Enablers to PITC services implementation

#### Program implementation strategy

PITC program information dissemination was one of the main enablers to implementation of PITC services. Although the national guideline was not distributed to TB-DOTS centers, an official letter was circulated by DPHO Kathmandu for mandatory uptake of HIV tests for registered TB patients in all TB-DOTS centers.

*“…. The hard copies might not have been supplied in service delivery points, however, the staff has been verbally and officially informed about guidelines during every orientation, training or meeting of TB program….” (Program manager 2, DPHO)*

#### Skilled and devoted human resources

Related training and orientation were also considered important enablers for program implementation. The service providers stated confidence in the uptake of HIV testing where HIV testing services were not available on the same site. Similarly, district managers were assured of effective implementation of PITC services by service providers of TB-DOTS centers.

*“…. The motivations of HWs can be boosted as they are provided with training as well as technical support and it’s helping in effective implementation of the program….” (Program manager 1, DPHO)*

#### Supervision and monitoring

Supportive supervision and monitoring from DPHO and NTC to service site were an important facilitator for service providers to get feedback and onsite coaching to improve their work at DOTS centers.

*“…. The TB program is effective itself and DPHO and NTC have been providing continuous supportive monitoring and supervision to implement the PITC service effectively and efficiently at service site….” (Health Workers 5, TB-DOTS)*

#### Resources and partnership

For effective implementation of PITC guidelines, the availability of resources, support from external development partners (EDPs), public-private partnership (PPP), and national program commitment were perceived as key enablers by program managers of DPHO.

*“…. The yearly program budget has been allocated for TB/HIV management from the central level and organizations working in TB/HIV field are also supporting to implement PITC program….” (Program manager 1, DPHO)*

### Barriers to PITC services implementation

#### Insufficient guideline dissemination and executive issues

According to health workers of the TB-DOTS centers, the national PITC guideline had not been received yet. Additioanlly, the health workers perceived the need for related training, especially for HIV services. A limited supply of rapid kits at TB-DOTS centers was considered as one of the significant barriers to service implementation.

*“…. Have not received the national PITC implementation guideline yet ….” (Health workers 4, TB-DOTS)*

*“.... As KIT was stock out, so we missed testing of some patients and also children HIV screen is not done….” (Health worker 2, TB-DOTS)*

#### Factors influencing patients’ acceptance of HIV testing

The service providers of TB-DOTS centers B and C perceived that patients’ self-stigmatization, patients’ self-trust, feeling of no exposure to the risk of HIV, and low acceptance of HIV towards children and aged patients were key barriers from patients’ views. Some patients initially accepted the HIV testing offer; however, they did not attend the test because of fear of knowing the result.

*“…. they are self-stigmatized by the concept if they have to perform the serological HIV test. The patients are reluctant at first if they have to do HIV test. Sometimes patients are angry and decline the offer of HIV screening directly. It has happened when the TB patients are a child, elder, and adult who is educated ….” (Health worker 3, TB-DOTS).*

According to the service provider of TB-DOTS center C, knowledge of several patients on HIV was quite good and they belong to the upper socio-economic status so patients were reluctant to accept the offered HIV test.

“…. *majorly the educated and rich family rejected to attend the HIV test because they know the way of HIV transmission and said they haven’t been exposed to risk behaviours. So, they say there is no need to check their HIV status and only ask for TB regimens as they are diagnosed with TB.….” (Health worker 3, TB-DOTS).*

#### Issues experienced by service providers

While most of the service providers faced significant challenges unaddressed by DPHO which includes lack of basic and refresher training related to TB/HIV, inadequate consultation space in service sites was also influencing proper implementation of PITC program in the selected DOTS centers.

*“…. Previously, DHO used to send us commodities after the completion of each quarter. Now, the problem is we have to go to get HIV test kit for ourselves, and I am the only staff here, which might disrupt services….” (Health worker 2, TB-DOTS)*

*“…. There isn’t a separate counselling room for patients, we manage a separate place (bench) for it….” (Health workers 1, TB-DOTS)*

#### Perceptions of program managers

Barriers experienced by program managers included being unable to provide training to all health workers and regularize the HIV testing at every health facilities.

*“…. We haven’t been able to regularize and make mandatory HIV test in all the districts, as we are not able to provide training to all the HWs….” (Program manager 1, DPHO)*

*“….Supply of HIV testing kits did not reach every service delivery point. Even if the test Kits are available at service delivery points, staff are not capable to perform the test….” (Program manager 2, DPHO)*

There were communication problems between service providers and TB patients mainly in cases of children and elderly, while lack of properly trained health workers and patients’ self-stigmatization were major barriers at the service site level.

*“…. from a patient’s perspective, it is a contrasting scenario. An HIV positive patient is willingly ready to give sputum for TB test, but the scenario is different in TB patients as they are not ready for HIV test, maybe HIV has always been stigmatized by the society….” (Program manager 1, DPHO)*

*“…. as from the HWs perspective, they find it difficult in convincing the TB patients to perform HIV tests. As suggested by them, HIV test for TB patients should be mandatory to an age group who are more susceptible. So it’s better to perform the HIV test in cases of treatment failure, regular positive results in follow up, prone age group or occupation history rather than to children and elderly people….” (Program manager 1, DPHO)*

Two different focal persons handle the TB and HIV programs in the Kathmandu district. Causing some preventable difficulty, there was only limited communication between each other regarding coordination and implementation of the TB/HIV programs in the district.

*“…. The focal person for TB and HIV are two different persons and are guided by their respective sections and centers making it difficult to work in cohesion, although the guideline spells out that it should be looked after by the same person….” (Program manager 2, DPHO)*

#### Resource limitations

Resource limitations were key barriers for program managers more than service providers. Insufficient budget for training and late disbursement of allocated budget were the main reasons for delayed implementation of targeted activities in the district.

*“…. We have not been able to provide training to all health workers due to the lack of budget….” (Program manager 1, DPHO)*

*“…. untimely disbursement of budget resulting in the late implementation of activities. Social and political context is always a challenge. In terms of logistics, we receive medicines and toolkits that are near expiration date….” (Program manager 2, DPHO)*

## Discussion

TB/HIV co-infection is a main opportunistic infection among both TB patients and PLHIV patients in developing nations [3]. Active detection of HIV infection among TB patients is necessary for direction to scale up ARV and special treatment approaches for TB patients [16].

### Outcome of PITC services

This study represents one of the first attempts in Nepal to assess the implementation fidelity of PITC among TB patients in the national TB control program. From the total of 643 TB patients registered in the care cascade, 591 (92%) patients were offered HIV testing and counseling (HTC). Amongst those offered HTC, 571 (96.6%) accepted and 523 (91.5%) were tested. Based on these findings the uptake of HIV testing at TB-DOTS centers among TB patients in the selected sites is high (91.5%). This uptake of HIV tests among TB patients is high compared to the Kathmandu district (19.5%) and national level (17%) in the previous fiscal year of 2015/16 [10]. Our findings are similar to the earlier findings from Cameroon (94.7%) [6], Vizianagaram, India (85%) [7], South India (92.4%) [8], Ethiopia (95.7%) [17], and Nigeria (93.9%) [18] where PITC implementation at TB-DOTS centers is effective.

The key programmatic component of the PITC approach, uptake of HIV testing was conducted with concentrated outcomes, as reflected in the strong adherence as intended by the program with high exposure at every step of the PITC approach (Offered, Accepted and Attended) and followed by a positive trend in TB patients responsiveness in three out of five of the selected TB-DOTS centers.

### Quality delivery of PITC services in TB-DOTS centers

TB-DOTS centers’ service availability and readiness assessment for the quality of program delivery indicated that three out of the five TB-DOTS centers performed HTC at the sites and the other two referred for uptake of HIV testing at the nearest HTC center. In contrast, there was no national PITC guidelines available at service sites, and there was a lack of separate consultation rooms, lack of certain equipment and stock-outs of rapid HIV test kits in TB-DOTS centers. Similar suboptimal logistics have been found in the implementing of PITC in Zimbabwe and Namibia [19, 20]. Service providers cited inadequate staff to manage both service delivery and administration activities, and less supervision from DPHO. Meanwhile our study found only two out of five service providers were trained with TB/HIV related training [19, 21]. Patients and service providers used face masks during service seeking and providing counseling but this was different from the study done in Uganda, which found that many patients and staff did not wear facemasks [21]. Thus, without TB/HIV related training exposure, it is reflected in less adherence of PITC program guidelines at three out of the five selected TB-DOTS centres. The partial adherence in the dissemination of the national PITC guidelines and insufficient supply of rapid HIV test kits highlight the barriers in PITC services implementation at the study sites. Despite the high uptake of HIV testing, the implementation and usefulness of national PITC guidelines, along with barriers and negative opinions have an effect on the fidelity and adherence to proper implementation of national PITC guidelines and PITC services delivery.

### Barriers to uptake HIV testing

This study showed a lack of national PITC guidelines with insufficient dissemination and executive issues, patients’ self-stigmatization, patients’ self-trust, resulting in HIV testing offers rejected especially for children and aged patients, as well as a lack of skill among service providers towards provision of PITC services, difficult social and political context, and lack of adequate budget and late disbursement of allocated budget as the key barriers for delivery of PITC services at the selected TB-DOTS centers in Katmandu, Nepal. In particular, perception of no exposure to risk of HIV infection by patients, patients not providing consent in case of children and elderly patients, and difficulties to counsel educated and rich patients to uptake HIV test were similar difficulties perceived by services providers which were documented in earlier studies of Zimbabwe, Indonesia and Uganda [19, 21, 22]. However, in contrast to these findings, patients’ education was perceived positively associated with uptake HIV testing in a study done in Ethiopia [23].

### Enablers to implement PITC services

All TB-DOTS centers offered PITC services among registered TB cases (92%) with only the formal instruction of DPHO. This study found a very high level of acceptance to implement PITC services in routine TB programs by TB patients and service providers with a minimal additional workload which was similar to the results of studies done in India [7, 8]. The devotedness of service providers towards providing PITC services was also one of the key enablers assessed, despite the barriers they perceived to implement the PITC program such as, lack of national guidelines, lack of related training and offers rejected by child, elders and educated patients. These motivations and positive attitudes resulting in effective implementation of PITC services have also been detected in a Uganda setting [21].

One study limitation was that the review of the TB register for DOTS outlets was limited to five TB-DOTS centers which included tertiary/referral hospitals located in the capital city of the country. Accordingly, these results may not actually represent the situation of primary heath care settings. Thus, a further study including a mix of public and private health care settings will better depict the actual scenario of most districts in Nepal. Also, the perceptions of registered TB patients were not studied to assess their adherence towards PITC services. Our study gives a basic understanding of implementation fidelity towards PITC services under the TB control program in Kathmandu district. Additionally, the findings present an overview of TB-DOTS readiness and barriers which need to be addressed for efficient and effective implementation and enablers which supported the program integration. Finally and conclusively, the study was conducted within a routine TB control program, so that generalization can be considered to apply to most other districts in Nepal.

## Conclusion

Our study revealed that implementation of PITC services is good in three out of five DOTS centers, with high HIV testing acceptance by registered TB patients of the selected DOTS centers which can effectively determine HIV infected status among TB patients and provide support to reduce the burden of TB/HIV co-infection. The proportion of TB patient’s uptake of HTC among those registered patients offered was high in the total study population. The service availability and DOTS centers’ readiness are considered in the acceptable readiness category, however, the TB-DOTS centers were lacking PITC guidelines, separate rooms for consultation and had poor physical conditions. Besides those concerns, the qualitative findings of the study identified some barriers and enablers to the PITC service implementation. The key barriers were education and socio-economic level of patients, professional competence in three of the DOTS centers, and timely disbursement of the allocated budget. Overall, the study highlighted the need of providing a national guideline, and related training to service providers, while addressing the barriers and enablers for effective and efficient implementation of PITC programs at DOTS centers in Nepal.

## Additional files

Additional file 1: Data extraction tool. (DOCX 14 kb)

Additional file 2: Modified DOTS Center readiness assessment tool. (DOCX 31 kb)

Additional file 3: Key Informant Interview and Indepth Interview Guide. (DOCX 16 kb)

## Data Availability

Results of this study were based on the data presented in the manuscript. Approaches to data collection are mentioned in the methods section of the manuscript. Please contact the corresponding author for additional data availability on reasonable request.

## Abbreviations

ART: Antiretroviral Therapy
CNR: Case Notification Rate
DOTS: Directly Observed Treatment Short Course
HIV: Human Immunodeficiency Virus
HTC: HIV testing and counseling centre
IDI: In-depth Interview
KII: Key Informant Interview
NCASC: National Centre for AIDS and STD Control
NTC: National Tuberculosis Center
PITC: Provider-Initiated HIV Testing and Counselling
PLHIV: People Living With HIV
TB: Tuberculosis
WHO: World Health Organization
