# Supplementary material for: Implementation fidelity of provider-initiated HIV testing and counseling of tuberculosis patients under the National Tuberculosis Control Program in Kathmandu District of Nepal: an implementation research
Source: BMC Health Serv Res. 2019 Aug 2;19:543. doi: 10.1186/s12913-019-4343-3 (PMC6679426; doi:10.1186/s12913-019-4343-3)
Supplement: Supplementary file 1 — Data extraction tool. (DOCX 14 kb) [file 12913_2019_4343_MOESM1_ESM.docx]

## **Annex III. DOTS Register Data Assessment Checklist;**

| **Data extraction sheet from routine Tuberculosis Register of DOTS centre** | | | | | | | | | | | | | | |
| --- | --- | --- | --- | --- | --- | --- | --- | --- | --- | --- | --- | --- | --- | --- |
| **District:** | | | | | | | | | | | | | | |
| **Fiscal Year:** | | | | | | | | | | | | | | |
| SN | Patients characteristics | | | TB characteristics | | | PITC Services | | | | HIV +ve TB Patients | HIV +ve TB Patients on ART | Lab Results | Treatment Outcomes |
|  | Sex | Ethnicity Code | Age | Type of Disease | Previously Treated Patients | Treatment Categories | HIV Test Offered | HIV Test Accepted | HTC Attended | Result |  |  |  |  |
| 1 |  |  |  |  |  |  |  |  |  |  |  |  |  |  |
| 2 |  |  |  |  |  |  |  |  |  |  |  |  |  |  |
| 3 |  |  |  |  |  |  |  |  |  |  |  |  |  |  |
| 4 |  |  |  |  |  |  |  |  |  |  |  |  |  |  |
| 5 |  |  |  |  |  |  |  |  |  |  |  |  |  |  |
| 6 |  |  |  |  |  |  |  |  |  |  |  |  |  |  |
| 7 |  |  |  |  |  |  |  |  |  |  |  |  |  |  |
| 8 |  |  |  |  |  |  |  |  |  |  |  |  |  |  |
| 9 |  |  |  |  |  |  |  |  |  |  |  |  |  |  |
| 10 |  |  |  |  |  |  |  |  |  |  |  |  |  |  |
