# Supplementary material for: Implementation fidelity of provider-initiated HIV testing and counseling of tuberculosis patients under the National Tuberculosis Control Program in Kathmandu District of Nepal: an implementation research
Source: BMC Health Serv Res. 2019 Aug 2;19:543. doi: 10.1186/s12913-019-4343-3 (PMC6679426; doi:10.1186/s12913-019-4343-3)
Supplement: Supplementary file 2 — Modified DOTS Center readiness assessment tool. (DOCX 31 kb) [file 12913_2019_4343_MOESM2_ESM.docx]

**DOTS CENTER READINESS ASSESSMENT**

☞ The purpose of this tool is to assess the readiness of the District DOTS to assess the quality of PITC services; to assess functionality and performance of the DOTS staff.

☞ STEPS- Meet the DOTS In-charge and explain the purpose of the visit. Consult with other staff as appropriate.

Please circle the appropriate answer or fill in the blanks, use pen.

**Facility Identification:**

District: ………………………. Date of Visit: ………………………

Name of Facility: …………….......................

Type of Facility: ……………………............

Managing Authority: ……………………….

Conducted By: a) ……………………………………………………….

b) ……………………………………………………….

Participation from DOTS Center: ……………………………………….

**General Information:**

FIND THE IN-CHARGE OF THE DOTS, OR MOST SENIOR HEALTH WORKER RESPONSIBLE FOR DOTS SERVICES WHO IS PRESENT AT THE FACILITY. READ THE FOLLOWING GREETING:

Good day! My name is Randeep Kumar. We are here to conduct a survey for study entitled **“Implementation Fidelity of Provider Initiated HIV testing and counselling of Tuberculosis Patients under the TB Control Program in Nepal”**.

Now I will read a statement explaining the study.

This study is part of researcher Master’s thesis. Your facility was selected to participate in this study. We will be asking you questions about PITC services.

Neither your name nor that of any other health worker respondents participating in this study will be included in the dataset or in any report; however, there is a small chance that any of these respondents may be identified later. Still, we are asking for your help to ensure that the information we collect is accurate.

You may refuse to answer any question or choose to stop the interview at any time. However, we hope you will answer the questions, which will benefit the services you provide and the nation.

If there are questions for which someone else is the most appropriate person to provide the information, we would appreciate if you introduce us to that person to help us collect that information.

At this point, do you have any questions about the study? Do I have your agreement to proceed?

INTERVIEWER'S SIGNATURE INDICATING CONSENT OBTAINED DAY MONTH YEAR

MODULE 1: Readiness of Health Facility to provide services (on the day of visit)

| **Number** | **Question** | **Result** | | | |
| --- | --- | --- | --- | --- | --- |
| **Staffing** | | | | | |
| 100 | I have a few questions on staffing for this facility. Please tell me how many staff with each of the following qualifications are currently assigned to, employed by to this DOTS center. | Assigned/Employed (Including Part Time) | | | |
| 01 | Generalist (non-specialist) Medical Doctors | \|  \|  \| \| --- \| --- \| | | | |
| 02 | Paramedical Professionals (Health Assistant and Auxiliary Health Worker) | \|  \|  \| \| --- \| --- \| | | | |
| 03 | Nursing Professionals (Staff Nurse and Auxiliary Nurse Midwifery) | \|  \|  \| \| --- \| --- \| | | | |
| 04 | Laboratory Technicians | \|  \|  \| \| --- \| --- \| | | | |
| **Basic Amenities** | | | | | |
| 200 | Does this facility have an available and functional following items? | YES………………..1  NO…………………2 | | | |
| 01 | Power (electricity or solar) | 1 2 | | | |
| 02 | Running water source in facility premises | 1 2 | | | |
| 03 | Room with auditory and visual privacy for patient consultations | 1 2 | | | |
| 04 | Means of communication (Telephone/Cell phone) | 1 2 | | | |
| 05 | Toilet for clients | 1 2 | | | |
| 06 | On average, how many hours per day is this facility open? | Less than 4 hours………1  5 to 8 hours………….....2  Above 9 hours………….3 | | | |
| **Basic Equipment** | | | | | |
| 300 | Please tell me if the following basic equipment and supplies used in the provision of client services are available and functional in this facility today. | Available | | Functioning | |
|  |  | Yes | No | Yes | No |
| 01 | Adult Weighing Machine | 1 | 2 | 1 | 2 |
| 02 | Thermometer | 1 | 2 | 1 | 2 |
| 03 | BP Instrument | 1 | 2 | 1 | 2 |
| 04 | HIV Rapid Test Kit | 1 | 2 | 1 | 2 |

| **Standard Precautions for Prevention of Infections** | | | | | | |
| --- | --- | --- | --- | --- | --- | --- |
| 400 | Please tell me if the following resources/supplies used for infection control are available in the general outpatient area of this facility today. | Available | | | Functioning | |
|  |  | Yes | No | | Yes | No |
| 01 | Sterilization equipment (i.e. autoclave with energy source) | 1 | 2 | | 1 | 2 |
| 02 | Puncture proof container and burning pit | 1 | 2 | | 1 | 2 |
| 03 | Single use – standard disposable syringes | 1 | 2 | | 1 | 2 |
| 04 | Soap or hand disinfectant (alcohol + glycerine) | 1 | 2 | | 1 | 2 |
| 05 | Gloves | 1 | 2 | | 1 | 2 |
| 06 | Masks | 1 | 2 | | 1 | 2 |
| 07 | HF level guidelines for standard precautions | 1 | 2 | | 1 | 2 |
| **Information System** | | | | | | |
| 500 | Now I would like to ask you a few questions about recording and reporting for information system | Yes………….…1  No……………..2 | | | | |
| 1 | Did the health facility submit the HMIS report on time? (observe last month report) | 1 | | | 2 | |
| 2 | Did the health facility submit the LMIS report on time (7th day) to the District in the last trimester? (Observe) | 1 | | | 2 | |
| 3 | Does the health facility display up-to-date monthly service coverage information in a visible place? (observe) | 1 | | | 2 | |
| **Monitoring and Supervision** | | | | | | |
| 600 | When was the last time this facility received a supervision visit from the higher level (DTLO, DHO or other)? | This Month…………..1  In the last 3 Months….2  Above 3 Months ago...3  Don’t Know………...98 | | | | →600  →600 |
| 501 | During the supervision visit, did the supervisor assess the following? | Yes | | No | |  |
| 01 | Drug and Commodities stock out and expiry | 1 | | 2 | |  |
| 02 | Staff availability and training | 1 | | 2 | |  |
| 03 | Data completeness, quality, and timely reporting | 1 | | 2 | |  |

MODULE 1: Available Services

| **Provider-Initiated HIV Testing and Counselling** | | | |
| --- | --- | --- | --- |
| 700 | Does this facility offer provider-initiated HIV testing and counselling services? | Yes……………………1  No…………………….2 | |
| Ask to be shown the location in the facility where provider-initiated HIV testing and counselling services are provided. Find the person most knowledge about provider-initiated HIV testing and counselling services in the facility. Introduce yourself, explain the purpose of the survey and ask the following questions. | | | |
| 701 | Do you have the national provider-initiated HIV counselling and testing guidelines available in this facility today? | Yes……………………1  No…………………….2 | |
| 702 | Have you or any provider(s) of provider-initiated HIV/AIDS counselling and testing services: | Yes | No |
| 01 | Received any training in voluntary counselling and testing (VCT) in the last two years? | 1 | 2 |
| 02 | Received any training in HIV/AIDS prevention, care, and management for Tuberculosis patients in the last two years? | 1 | 2 |
| 703 | Does this facility provide HIV counselling and testing services to Tuberculosis patients? | 1 | 2 |
| 704 | Is the HIV testing and counselling service room or area a private room/area with auditory and visual privacy? | 1 | 2 |
| 705 | Do you have HIV rapid test kits (with valid expiration date) in stock in this service site today?  CHECK TO SEE IF VALID (NOT EXPIRED) | 1 | 2 |
| 706 | Do you have condoms available in this service site today to give to clients receiving services? | 1 | 2 |
