# Supplementary material for: Implementation fidelity of provider-initiated HIV testing and counseling of tuberculosis patients under the National Tuberculosis Control Program in Kathmandu District of Nepal: an implementation research
Source: BMC Health Serv Res. 2019 Aug 2;19:543. doi: 10.1186/s12913-019-4343-3 (PMC6679426; doi:10.1186/s12913-019-4343-3)
Supplement: Supplementary file 3 — Key Informant Interview and Indepth Interview Guide. (DOCX 16 kb) [file 12913_2019_4343_MOESM3_ESM.docx]

## **Annex VIII. In-depth Interview Guideline for Service Providers**

**A) General**

Your Sex: ……………………..

Your age (in years): …………………………………………..

Level of education: …………………………………………..

Your responsibility at the health facility: ………………………….

Your area of expertise: ………………………………….

Total years of service: …………………………

What types of settings have you previously worked in (ex: Hospital (Public/Private), Clinic (Public/Private), etc)? ................................................................

**B) PITC Intervention**

1. Can you tell me the objective of the PITC service delivery for TB patients at your setting?
2. From your experience, to what extent do you think the guidelines to PITC services are adequate enough to enhanced access and adherence to HIV-testing by the health workers and Tuberculosis patients?

***Probe:***

1. *Can you explain how?*
2. *How do you give information to patients about this program before enrolment?*
3. *What would you do if eligible patient refuse to be enrolled?*
4. *What can you suggest that could be considered in future efforts?*
5. What Operational Challenges have you been experiencing in the PITC services?

***Probe:***

1. *How frequently do you need to request for drugs and KITs from DHO and how do you maintain medicine stock at your Centre?*
2. *Have there been any cases that HIV testing and TB treatment has been interrupted due to stock out of medicine?*
3. *What are the criteria for follow up for patients who are under DOTS Centre?*
4. What enabling factors do you find in adopting the national PITC guideline?
5. Do you believe TB patients have a proper adherence to HIV-testing? If so, how do you think this have come to be? (If not)-What key barriers have you been observing patients have on HIV-testing adherence?

***Probe:***

1. *What is your experiences regarding acceptance of this method (PITC) by patients, where with this method, they risk being exposed to having another disease at same time?*
2. *What is your opinion regarding NTP’s commitment and efforts regarding making PITC services?*
3. How long have you been working as a Service Provider for PITC?
4. Have you participated in PITC, HTC and other TB/HIV service trainings?

***If Yes,***

1. *How often?*
2. *Where? Who trained you?*
3. How do you find of the benefits of the training?
4. Do you believe that the training you got is enough?

Thank you so much for providing your valuable time in this interview. I apologize if there were anything that offended you. If there is any other necessary information are you willing to be called back?

Thank you once again.

## **Annex IX. Key Informant Interview Guideline for Program Manager**

**A) General**

Your Sex: ………………………………………………

Your age (in years): ……………………………………

Level of education: …………………………………….

Designation: …………………………. ………………..

Your area of expertise: …………………………………

Total years of service: ………………………………….

**B) Policies and Guideline for PITC Activities**

1. How have you been dealing with TB/HIV program in this district?
2. Do you promote the use of national PITC guideline among DOTS centres in this district among health workers?

**Prove:**

*If yes, can you please explain how?*

*If not, can you please explain why?*

1. Can you please explain what are the mechanism for disseminating guideline is, in this district?
2. Do you have monitoring and feedback system after rolling out guidelines? Can you please explain the mechanism?
3. What barriers do you find for adherence to PITC guideline in your district?
4. What are the enabling factors in adhering to PITC guideline in your district?

Thank You
